# Vaginal Tumours, with Special Reference to Cancer and Sarcoma

**Published:** 1905-09

**Authors:** 


**Vaginal Tumours, with Special Reference to Cancer and Sarcoma.** By W. Roger Williams. Pp. viii., 92. London: John Bale, Sons and Danielsson Ltd. 1904.

This work forms a valuable contribution to the literature dealing with diseases of women. In a comparatively small space the author has succeeded in presenting a very lucid and complete account of the subject of the new formations met with in the vagina, with a clear and accurate account of the pathology of each variety.

He remarks the small liability of certain parts of the Müllerian system, notably the vagina and Fallopian tubes, to tumour formation when compared with other parts, such as the uterus. This, in his opinion, seems to show that functional peculiarities influence tumour formation more than the developmental origin of the organ involved. He points out that the rare incidence of cancer in the vagina is rather against the irritative theory of its production, and shows that it is rare in prostitutes, and also in connection with either the wearing of pessaries or in connection with prolapse and invagination. He gives a very interesting account of vaginal cysts, especially as to the production of cysts of the Wolffian ducts and also those derived from the Mullerian system.

We can recommend a careful study of this work to anyone who wishes to gain information on the subject. The numerous references make the consultation of the various authorities quoted an easy matter.

